# Report on the FEBS‐IUBMB‐ENABLE 1st International Molecular Biosciences PhD and Postdoc Conference

**DOI:** 10.1002/2211-5463.13609

**Published:** 2023-06-16

**Authors:** Marta Reyes‐Corral, Maren Pfirrmann

**Affiliations:** ^1^ Institute of Biomedicine of Seville (IBiS) / Virgen del Rocío University Hospital / CSIC / University of Seville Seville Spain; ^2^ Radboud Institute for Molecular Life Sciences (RIMLS) Nijmegen The Netherlands

**Keywords:** Conference report, ENABLE, FEBS, IUBMB

## Abstract

The FEBS‐IUBMB‐ENABLE 1st International Molecular Biosciences PhD and Postdoc Conference was held in Seville, Spain, from the 16–18th of November 2022. Nearly 300 participants from all over the globe were welcomed by the host institution, the Institute of Biomedicine of Seville (IBiS). Following the theme “The perfect tandem: How technology expands the frontiers of biomedicine”, the Scientific Symposium of the conference hosted eight world‐renowned keynote speakers who presented their work in one of the four sessions: *Innovation*, *Basic Research*, *Translational and Clinical Research*, and *Computational Biology and Artificial Intelligence*. Participants had the chance to present their research to their peers: more than 200 posters were presented during the dedicated poster sessions and 19 selected PhD students and postdocs presented their work as short talks. The Career Day featured a wide range of workshops fully devoted to trainees' professional development, as well as a job fair and career chats with professionals to discuss future perspectives. Besides, several outreach activities were organised before and during the conference to engage with the general public and bring science closer to society. The success of this conference will be followed by the next FEBS‐IUBMB‐ENABLE conferences in Cologne, Germany, in 2023 and Singapore in 2024.

## Graphical abstract

The FEBS‐IUBMB‐ENABLE 1st International Molecular Biosciences PhD and Postdoc Conference was held at the Institute of Biomedicine of Seville in Spain, from 16–18th November 2022. Following the theme “The perfect tandem: How technology expands the frontiers of biomedicine”, the conference gathered 300 participants from all over the globe and included a Scientific Symposium, a Career Day, and outreach activities.

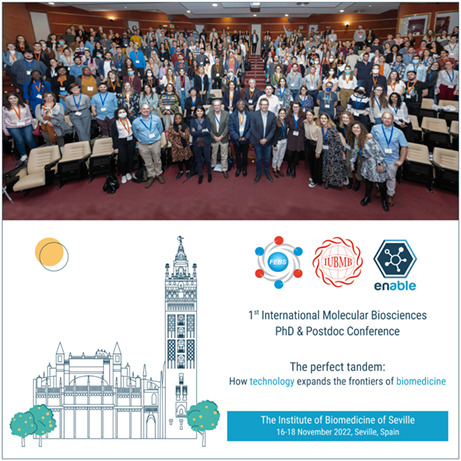

**Abbreviations**
CPR: Novo Nordisk Foundation Center for Protein Research
ENABLE: European Academy for Biomedical Science
FEBS: Federation of European Biochemical Societies
IBiS: Institute of Biomedicine of Seville
IRB: Institute for Research in Biomedicine
IUBMB: International Union of Biochemistry and Molecular Biology
RIMLS: Radboud Institute for Molecular Life Sciences
SEMM: European School of Molecular Medicine

Originally supported by funding from the European Union Horizon 2020 initiative, ENABLE (the European Academy for Biomedical Science) began as a collaboration between four renowned European research institutes: the Institute for Research in Biomedicine – IRB (Barcelona, Spain), the Radboud Institute for Molecular Life Sciences – RIMLS (Nijmegen, The Netherlands), the Novo Nordisk Foundation Center for Protein Research – CPR (Copenhagen, Denmark), and the European School of Molecular Medicine – SEMM (Milan, Italy), together with Scienseed, a science communication agency.

ENABLE started as an initiative to promote European excellence in biomedical science with an annual three‐day international event that included a scientific symposium, career development sessions, and outreach activities. After the initial funding for ENABLE ended, the Federation of European Biochemical Societies (FEBS) and the International Union of Biochemistry and Molecular Biology (IUBMB) – which sponsored all ENABLE conferences – partnered to continue this initiative and support the development of young researchers.

This year's conference took place in the beautiful and sunny city of Seville, the capital of the amazing southern region of Andalusia, in Spain. The host institution of the conference was the Institute of Biomedicine of Seville (IBiS), a multidisciplinary biomedical research centre created in 2006 and located within the complex that houses the Virgen del Rocío University Hospital, a centre of high‐level care, education, and research.

The entire conference – from scientific sessions to social events – was organised by a group of young and enthusiastic scientists. The Scientific Organising Committee was an international group that included pairs of representatives from the host institution (IBiS) and the four collaborating research centres (IRB, RIMLS, CPR, and SEMM), as well as one representative from FEBS and another from IUBMB. The Local Organising Committee consisted of 24 PhD students and postdocs from the host institution and was responsible for organising the social programme, the Career Day activities, and all of the conference logistics. In addition to this, the Scientific and Local Organising Committees had the support of coordinators from the five research institutions as well as members of FEBS and IUBMB (Table 1).

**Table 1:** List of organisers of the FEBS‐IUBMB‐ENABLE 2022 conference.

Scientific Organising Committee (SOC)		Coordinators		Local Organising Committee (LOC)	
Marta Reyes Corral (*SOC Chair*)	IBiS	María Herreras Casado	IBiS	Aida Amador Álvarez	Esperanza Lisha Granado de la Calle
Marina Esteban Medina	IBiS	María D. Giráldez	IBiS	Francisco José Calero Castro	Antonio Jurado Gómez‐Alférez
Olga Roman	IRB	Olivia Tort	IRB	David Carneros Trujillo	María Isabel Lara Chica
Camilla Bertani	IRB	Leyre Caracuel	IRB	Cynthia Clemente González	Macarena López Sánchez
Charu Jain	CPR	Moreno Papetti	CPR	Hildegard Colino Lage	Marta Martín Bórnez
Rosa Lundbye Allesøe	CPR	Marie Sofie Yoo Tollenaere‐Larsen	CPR	Laura Contreras Bernal	Sara Martín Villanueva
Maren Pfirrmann (*SOC Vice‐Chair*)	RIMLS	Clasien Oomen	RIMLS	Patricia de la Cruz Ojeda	Emilio Martínez Márquez
Javier Botey Bataller	RIMLS	Francesca Fiore	SEMM	Carmen Del Río Mercado	Esperanza Muñoz Muela
Erica Gasparotto	SEMM	Irene Díaz Moreno	FEBS	Sara Fontalva Ostio	Alberto Pérez Gómez
Martina Pezzali	SEMM	Jerka Dumić	FEBS	Vanesa Garrido Rodríguez	Eduardo Ponce España
Vlastimil Kulda	FEBS	Ilona Concha Grabinger	IUBMB	Mónica González Moreno	Diana Rubio Contreras
Daniel Dries	IUBMB	Lim Yang Mooi	IUBMB	Carmen María Gordillo Vázquez	Antonio Jesús Tagua Jáñez

With the title “The perfect tandem: How technology expands the frontiers of biomedicine”, this year's conference was all about the influence of technology on the field of biomedicine. Bringing together newly developed technologies with biomedical research can lead to remarkable discoveries. Therefore, the conference aimed to encourage young researchers to embrace technological innovations to foster scientific progress. To highlight the latest discoveries, eight keynote speakers were invited to present their interpretation of “The perfect tandem” in one of the four different sessions: *Innovation*, *Basic Research*, *Translational and Clinical Research*, and *Computational Biology and Artificial Intelligence*. The speakers were selected for their outstanding work in their respective field by showing how technology helped their research to move forward.

Nearly 300 participants joined the 1st FEBS‐IUBMB‐ENABLE Conference in Seville, making this conference the biggest in‐person event in the history of ENABLE. Participants – mostly PhD students and postdoctoral researchers – joined from 31 different countries, representing over 40 different nationalities (Fig. 1), to be part of a conference devised for young scientists to encourage their scientific career. Participants could not only join a scientific symposium with international renowned speakers but could also attend workshops and career chats tailored for their specific needs. To encourage young scientists also means to support them no matter where they come from and to help provide access to events like this to all people equally. With the support of our ten sponsors, a total of 74 travel grants were awarded to excellent young scientists to join our conference. The travel grants allowed many applicants from economically challenged countries to participate and finance their trip. To make the conference more accessible to applicants from outside of Europe, eleven overseas applicants were supported with a travel grant. Additionally, IUBMB awarded five travel grants to African applicants and the Croatian Society of Biochemistry and Molecular Biology (HDBMB) awarded two travel grants to its junior members. Overall, 50% of participants who applied for a travel grant were awarded one.

**Fig. 1:**
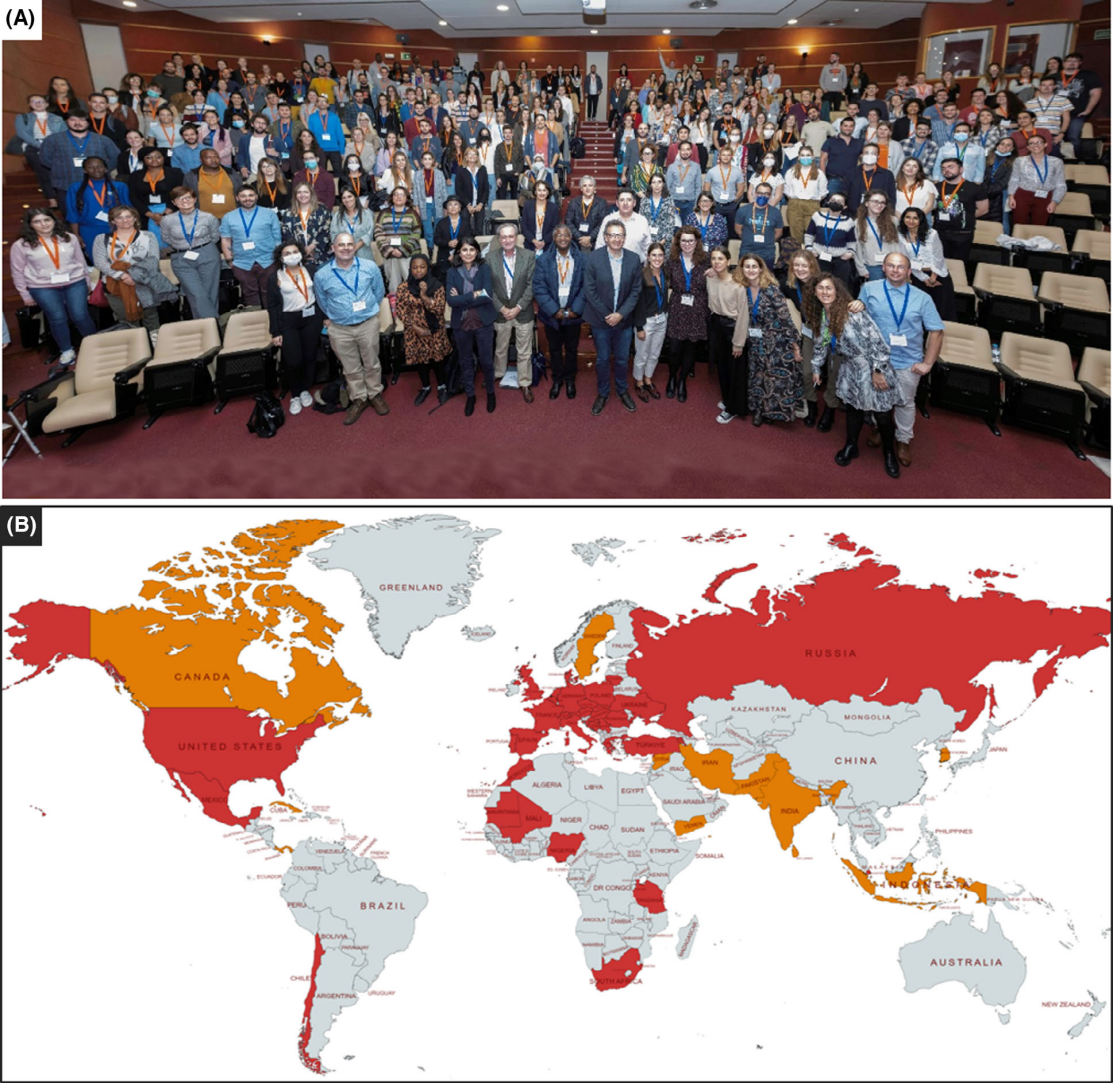
Participants of the 1st FEBS‐IUBMB‐ENABLE PhD and Postdoc Conference. (A) Nearly 300 participants gathered in Seville from all over the world to join the FEBS‐IUBMB‐ENABLE Conference. (B) This map represents the different countries of origin of the participants (the countries in which their research institutions are located), highlighted in red. The nationalities of participants not included in the previous list are highlighted in orange.

With an enthusiastic organising team, 300 participants from all over the world, excellent keynote speakers and three days full of science and fun, the 1st FEBS‐IUBMB‐ENABLE Conference in Seville took place as follows.

## Day 0 – A social night to get to know each other

A series of social activities for the evening before the conference officially started were organised by the Local Organising Committee, to allow participants to get to know each other and to discover the city of Seville. These optional activities were a huge success: over 150 participants gathered at Casa de la Ciencia, the Science Museum of Seville, to pick up their registration bags and start meeting each other. This was followed by a free guided tour around some of the most historical monuments of the city, such as Plaza de España or the Cathedral, that everybody very much enjoyed despite some unfortunate (and infrequent!) light rain. The highlight of the night was a private tour to the Real Alcázar, the Royal Moorish Palace of Seville and an UNESCO World Heritage site. To finish a great night, the participants enjoyed some drinks and fun games, such as social bingo and quizzes, in the popular nightclub district of Alameda.

## Day 1 – The conference begins! First day of the scientific symposium

Day 1 began with a welcome from the various organisers of the conference, including a welcome speech from Prof. Rafael Fernández‐Chacón, director of IBiS. The scientific symposium was organised into four sessions. In each session, two distinguished keynote speakers presented their research and also gave an overview of their scientific career. Each keynote lecture was followed by three short talks presented by outstanding PhD students and postdocs chosen from among the 300 participants. Moreover, over 200 posters from a wide range of biomedical fields were presented during the four scheduled poster sessions, facilitating vibrant discussion between young researchers with diverse backgrounds.

Prof. Matteo Iannacone from the San Raffaele Scientific Institute in Milan (Italy) opened the first session of the day, *Innovation*, and presented his work on the immune response to hepatitis B virus and SARS‐CoV‐2. Three selected short talks given by José David Celdrán‐López, Anna Georgina Kopasz, and Viktoriia Gross followed his lecture. Afterwards, the first poster session of the symposium took place at the patio of Virgen del Rocío University Hospital, in which the participants could share their research with their peers in a relaxed atmosphere while enjoying some coffee and pastries.

The session on *Computational Biology and Artificial Intelligence* followed with two scientific short talks from Katerina‐Marina Pilala and John Omo‐Osagie Uhomoibhi. Due to Covid‐19 restrictions, Prof. Elena Papaleo (Technical University of Denmark) could not join the conference and present her keynote lecture. Before the lunch break, David Cano‐González, PI at IBiS, raised awareness for the dangers of pseudoscience and misinformation in his science outreach talk. After a morning full of science, the participants went to lunch, and were able to enjoy some warm Spanish sun after the previous day's unfortunate weather.

Prof. José López‐Barneo, former director of IBiS, opened the afternoon session on *Basic Research* with a keynote lecture about his work on acute oxygen sensing by arterial chemoreceptors. Barbora Smolková, Luis Flores Horgue, and Nora Hase followed his keynote lecture, presenting their contribution to the field of basic research. Before the last session of the day, participants enjoyed a second session of posters.

The last session of the day, *Clinical and Translational Biomedicine*, started with a keynote lecture given by Prof. Fathia Mami‐Chouaïb from INSERM Gustave Roussy Institute (Villejuif, France). During her talk, she highlighted the importance of CD8 T cells in oncoimmunology. Three short talks from Mariano Molina, Thuy Nguyen, and Sikozile Ncembu closed the first day of the scientific symposium.

However, the participants that still wanted more science could join the *Science in the pubs night* planned by the Local Organising Committee. Participants were given the chance to present their work in a more informal setting while sharing some drinks. Similar to an elevator pitch, 11 young scientists introduced their research in just 3 min to the audience, and then all attendees voted for their favourite presentation. This fun activity closed Day 1 of the Scientific Symposium.

## Day 2 – Second day of the scientific symposium: Talks, posters, gala dinner, and more!

Day 2 of the scientific symposium followed a similar structure and started off with the second session on *Computational Biology and Artificial Intelligence*. Prof. Joaquín Dopazo‐Blázquez, from IBiS/Fundación Progreso y Salud (Seville, Spain), gave a keynote lecture online about repurposing drugs for rare diseases using machine learning and big data. Three short talks in this field were given by Daniel Martín, Terezie Páníková, and Aikaterini Despoina Nastou.

After the mid‐day poster session and some coffee to recharge and chat to peers, the session on *Clinical and Translational Biomedicine* began with Dr. Laura Cancedda from the Italian Institute of Technology (Genoa, Italy). Her talk guided the audience through the long and winding road of biomedical research towards treating neurodevelopmental disorders. Her inspirational story showed the first steps of her research leading to a start‐up company and clinical trials. Next, Catarina Pimpão and Cosmin Trif presented their work in two short talks. Francisco José Román‐Rodríguez closed the morning session with his scientific monologue on the “Journey to the centre of the cell” as part of the science outreach programme.

The afternoon session was opened by Dr. Daphne Cabianca from the Institute of Functional Epigenetics (Munich, Germany), with her keynote lecture in the session on *Basic Research*. She presented her work on understanding the spatial genome architecture by developmental and environmental cues. The last three short talks of the symposium followed, and were given by Ana‐Maria Pantazica, Oskar Ciesielski, and Carmen Torres‐Granados.

As the Scientific Symposium was coming to an end, participants enjoyed the fourth and last poster session. Prof. César de la Fuente from the University of Pennsylvania (USA) gave the last keynote lecture of the symposium in the session *Innovation*. He introduced his work on artificial intelligence approaches for the discovery of new antibiotics.

The last activity of the Scientific Symposium was a round table on *Open Science* with the focus on open access publishing. The moderators Irene Díaz‐Moreno and Jerka Dumić (FEBS) welcomed the panel composed by Dirk van Gorp (Open Science Officer at Radboud University), Valerie Teng‐Brough (Senior editor at Elsevier), Eleni Skourti (Editor at The FEBS Journal) and two of the keynote speakers: Profs. José López‐Barneo and Fathia Mami‐Chouaïb. After a small introduction to the topic by Dirk van Gorp, the audience was able to vote on different topics to be discussed by the panel. Besides the costs of open science, the discussion also included the issue of publication accessibility for developing countries and the impact of open access on science itself. The audience actively engaged in a conversation with the panellists, who shared their different perspectives about open science as academics and editors.

To finish off Day 2 of the conference and with that also the scientific symposium, all the participants were invited to a Gala Dinner at a beautiful restaurant by the river Guadalquivir. Although the weather did not play along, the three‐course meal made up for the rain and fog. Along with good food, everyone could relax and connect with the other participants. This moment was also used to thank the organisers and everyone involved in making this event happen. After the dinner, the night continued until late in a nearby bar with some good music, before everyone got some rest for the last day of the conference.

## Day 3 – Career day and goodbye until next year

After two intense days filled with science, the third and last day of the conference, known as the Career Day, started with a dynamic round table on *Transferable skills for alternative careers in Science*. In this round table, several of the keynote speakers from the scientific symposium (Profs. César de la Fuente, Matteo Iannacone, and José López‐Barneo) had an engaging discussion with other academic and non‐academic scientists (Ferhan Sağın, FEBS; Joan Guinovart, IRB; and Rebeca Kenda Nana Barrantes, Libertad con Ciencia), a conversation that was moderated by Jerka Dumić (FEBS).

The morning programme included several “How‐to” workshops on various topics: “How to build your own start‐up” by José A. Horcajadas (CEO of several technological companies), “How to write a research paper” by Valerie Teng‐Broug (senior publisher at Elsevier), and “How to create a culture of sustainability in science” by Carlo Battisti (My Green Lab Ambassador). In addition, Manolo Castellano (Career Consultant and Talent Recruiter, Carreras Científicas Alternativas) gave a seminar on “Alternative careers for scientists and researchers” and Prof. Luisma Escudero (University of Seville/IBiS) gave some tips for those interested in science outreach in his workshop “Can you improve your presentation skills?”

**Fig. 2:**
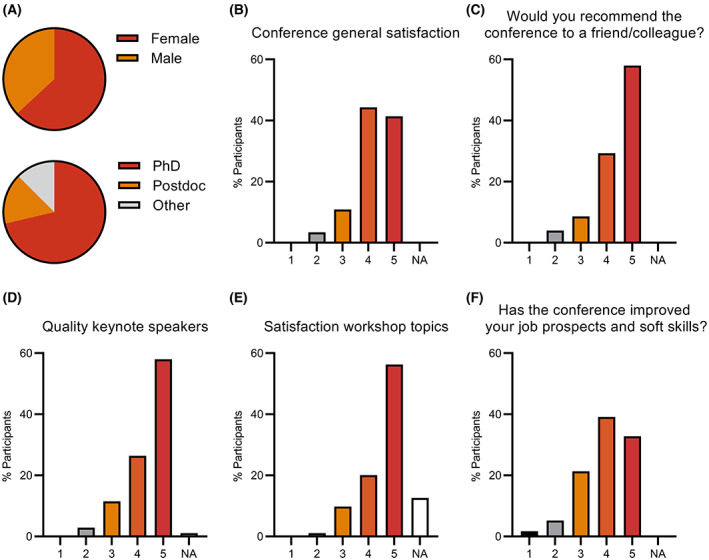
An insight into the participants of FEBS‐IUBMB‐ENABLE and their satisfaction with the conference. (A) Demographic data from the conference participants. (B–F) Results from an anonymous satisfaction survey conducted online after the conference, *n* = 174 responses.

The afternoon programme continued with some more “How‐to” workshops: “How to elaborate a cogent grant application… to get it funded!” by Prof. Miguel A. de la Rosa (University of Seville and FEBS Secretary General) and “How to land a job in industry (even if you aren't ‘qualified’)” by Rebeca Kenda Nana Barrantes (Associate Consultant and Career Coach, Libertad con Ciencia). The session also included workshops on transferable skills: “Effective networking inside and outside academia” by Manolo Castellano (Carreras Científicas Alternativas) and “Keys to succeed in a job interview” by Sašo Kočevar (Founder and Director of HFP Consulting) as well as some workshops on career paths: “Create your professional identity and gain clarity on your career path” by Amani Said (Career Coach for scientists) and “Shaping your career also as an educator – Tips and tricks for a young scientist” by Prof. Ferhan G. Sağın (Ege University and Chair of the FEBS Education Committee). Overall, the workshops of the Career Day were one of the activities of the conference that the participants enjoyed the most: 76% of the participants said that they were satisfied/very satisfied with the workshop topics and 72% agree/strongly agree that participating in FEBS‐IUBMB‐ENABLE has helped them improve their job prospects and soft skills (Fig. 2).

Besides the workshops, participants also had the opportunity to discuss various career paths with professionals from different fields. During the morning session, Joan Guinovart (Emeritus Professor at IRB), Yasin Hanaee (Director and CEO of COBIOMIC), and Mirjam Brullemans (Data Steward at Radboudumc Technology Center Data Stewardship) shared their expertise and invited participants to ask questions. In the afternoon session, José A. Horcajadas (CEO of several technological companies), Clara Caminal (Senior Manager, EU Project Office, RMIT Europe), and Valerie Teng‐Broug (Senior publisher, Elsevier) joined the tables to discuss their respective career paths with participants and give their advice and tips to young researchers. To wind up the Career Day, an opportunity fair was set up in the patio of the hospital building with stands from local companies and supporting universities, as well as representatives of FEBS and IUBMB who informed the participants about the initiatives for young scientists.

After all the workshops and career chats, the participants were invited to join the closing ceremony. Prof. Joan Guinovart (IRB), past President of IUBMB and under whose leadership ENABLE began, gave an overview of the history of the past ENABLE conferences. Afterwards, Prof. Irene Díaz‐Moreno (Chair of the FEBS Working Group on the Careers of Young Scientists) talked about the future of FEBS‐IUBMB‐ENABLE, revealing that the conference series will continue due to its great success. Vlastimil Kulda (FEBS) followed her by introducing other FEBS initiatives for young scientists, including the Young Scientists Forum, and the FEBS Junior Section also had a chance to present their initiative. Finally, the 2nd FEBS‐IUBMB‐ENABLE Conference, which will take place in Cologne (Germany) in November 2023, was introduced by Franziska Baumann and Andrea Mariani, chair and vice‐chair of the Scientific Organising Committee 2023.

Most importantly, the ceremony also included an announcement of all the prizes that participants won for their excellent work. A total of 14 prizes were given. The first three prizes were given out for the scientific photography contest, which was evaluated by an international expert panel. This was followed by the prizes for the best poster, with a total of eight prizes (two per poster session). Some of these awards were kindly sponsored by FEBS and IUBMB journals: *FEBS Open Bio*, *IUBMB Life*, *IUBMB BioFactors*, and *Biotechnology and Applied Biochemistry*. The award ceremony ended with the prizes for the best short talk presentations. The first prize, sponsored by Biomol, was given to Luis Flores‐Horgue, the second prize, sponsored by Olink, to Mariano Molina, and the third prize to Aikaterini Despoina Nastou.

**Fig. 3:**
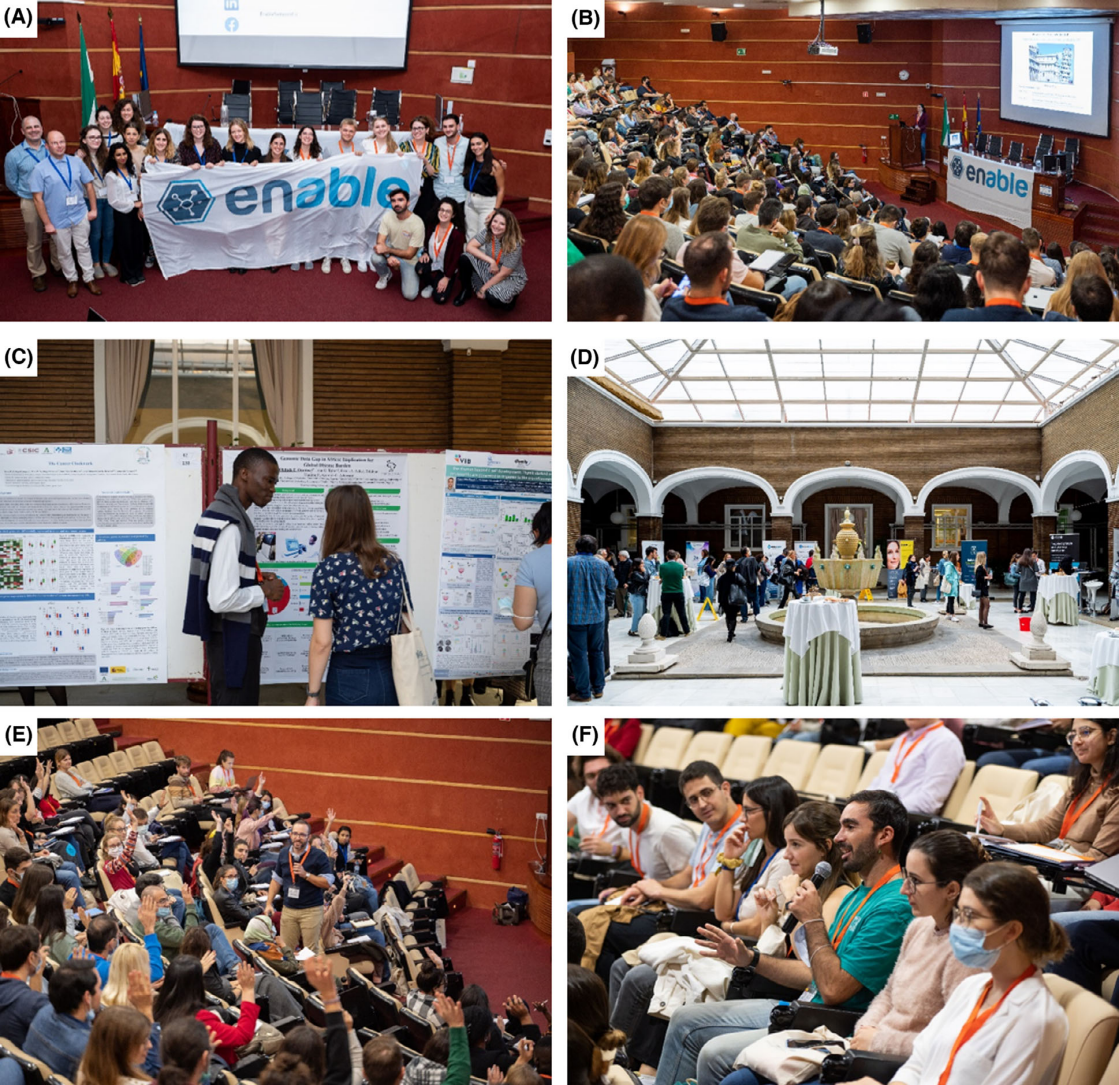
A glimpse of the FEBS‐IUBMB‐ENABLE Conference. (A) Members of the Scientific Organising Committees of Seville 2022 and Cologne 2023 conferences. (B) Keynote speaker Dr. Laura Cancedda explaining her research during the *Clinical and Translational Biomedicine* session of the Scientific Symposium. (C) Participants sharing their research during one of the four poster sessions. (D) Job fair organised in the patio of Virgen del Rocío University Hospital as part of the Career Day activities. (E) Participants engaging in one of the career development workshops. (F) Interactive discussion from the audience during the round tables.

The only thing left were the final words from the Chair of the Scientific Organising Committee, Marta Reyes‐Corral. She thanked all the participants for their attendance and all the helping hands for making the conference the success it was. As her final duty, she handed over the flag of ENABLE to Franziska Baumann, the Chair of the next Scientific Organising Committee, thereby closing the 1st FEBS‐IUBMB‐ENABLE 2022 Conference in Seville (Fig. 3).

## Outside the conference – Bringing science closer to society

The ENABLE conference is more than just a conference. Traditionally, the conference also includes several outreach activities to engage with the general public and bring science closer to society. This year's outreach activities started one week before the conference with *Semana de la Ciencia* or the Week of Science. In collaboration with the University of Seville and associated institutions, several public engagement events were organised during *Semana de la Ciencia*. Two scientific talks were given to secondary school students by researchers from the initiative *Ciencia a la Carta*. This included the talks “PCR, much more than COVID diagnosis” and “The risk of unhealthy eating: neurodegenerative diseases and nutrition”.

Additionally, secondary school students were invited to visit the host institution, IBiS, where they were able to meet researchers from different fields, learn what it means to be a scientist, and see how experiments are performed in the labs. Besides, a group of young researchers from IBiS also organised the outreach activity “What is a PhD?”. In a conversation with citizens from a nearby Sevillian neighbourhood, IBiS researchers shed some light on and uncovered some of the myths surrounding this crucial step in the life of a scientist.

Finally, there were some more outreach activities organised during the conference for its participants. During the “Science in the pubs night”, the participants faced the challenge of explaining their PhD or postdoc projects to their peers – from very different fields – in just 3 min. They did a great job in explaining their research, with all its depth and complexity, in a relaxed and entertaining atmosphere of being in a pub with friends. Also, because a picture is worth more than a thousand words, or so the saying goes, a scientific photo contest was organised, in which participants could show the product of their research summed up in one picture. The photos submitted to this contest were displayed in the hall of the Virgen del Rocío University Hospital building where the congress took place, so not only the conference participants but also the medical students and health professionals who work there could get a glance of what scientists do.

## What comes next?

After the success of the 1st FEBS‐IUBMB‐ENABLE Conference in Seville, there can only be one conclusion: more conferences like this need to come!

Thanks to FEBS and IUBMB, the conference series will continue. The 2nd FEBS‐IUBMB‐ENABLE Conference is currently being organised and will be held at the University of Cologne, in Germany. This upcoming conference will for the first time highlight the importance of the environment on human health under the motto #enablegoesgreen! Eight keynote speakers have already been invited to share their knowledge in their respective field of research. The new Scientific and Local Organising Committees are working on an exciting programme to allow young researchers to connect and share their science. With the title “The emerging challenge – Environmental impacts on human health”, the conference will be held on 23–25th of November 2023. For more information, please visit the ENABLE website (https://febs-iubmb-enableconference.org/).

The 3rd FEBS‐IUBMB‐ENABLE Conference in 2024 will be held in a non‐European country for the first time in the history of ENABLE. The Lee Kong Chian School of Medicine – Nanyang Technological University, Singapore, will be the host of the 2024 conference and will welcome participants to join another international conference devoted to PhD students and postdocs. We hope that the community of young scientists that met in Seville will continue to grow through the future FEBS‐IUBMB‐ENABLE conference series!

